# Measures to Contain the Transmission of Hepatitis C in a Chronic Kidney Care Hospital Unit in the Triângulo Mineiro in Brazil: A Case Study

**DOI:** 10.3389/ijph.2023.1605914

**Published:** 2023-05-31

**Authors:** Geisa Perez Medina Gomide, Lívia Helena de Morais Pereira, Fernanda Carolina Camargo, Lara Maximiano Rodrigues, Regiane da Silva Souza, Isadora Vieira de Melo, Thayná Andreza Ribeiro Pereira, Cristina da Cunha Hueb Barata de Oliveira

**Affiliations:** ^1^ Department of Internal Medicine, Federal University of Triângulo Mineiro, Uberaba, Brazil; ^2^ Urinary System Unit, Hospital de Clínicas, Federal University of Triângulo Mineiro, Uberaba, Brazil; ^3^ Teaching and Research Management, Hospital de Clínicas, Federal University of Triângulo Mineiro, Uberaba, Brazil; ^4^ Federal University of Triângulo Mineiro, Uberaba, Brazil

**Keywords:** hepatitis C, epidemiology, public health services, hemodialysis units, hospital, patient care team

## Abstract

**Objectives:** Hepatitis C virus elimination is complex. The objective was to analyze measures to eliminate virus transmission in a hemodialysis unit.

**Methods:** Case study composed of multiple units of analysis. The scenario is the hemodialysis unit of a Brazilian public hospital. Population composed of health service records. Descriptive analyzes were performed and the beginning of the event was considered as the moment of increased incidence of HCV. The intentional and purposeful collection of information for understanding the event and implementing interventions.

**Results:** The subunits of analysis were related to: clinical-epidemiological profile, active search, transmission routes, management protocol and results achieved. In August 2019, out of 45 patients, six were reactive for anti-HCV. All received treatment. Patients had exposure to contaminated medical equipment, objects or hands of professionals. Preventive measures were adopted and routine techniques were corrected. Situational Analysis Committee guided the management of the event. No new cases were detected.

**Conclusions:** Strategies for the microelimination of the C virus in a dialysis environment are demonstrated and it shows the multidisciplinary efforts in conducting the event.

## Introduction

Worldwide Hepatitis B and C virus infections are the main causes of chronic liver disease, cirrhosis, and hepatocellular carcinoma. Complications resulting from viral hepatitis and the disease itself represent a public health challenge for the Unified Health System (SUS). The United Nations (UN) Sustainable Development Goals (SDGs) include: “ending the epidemics of AIDS, tuberculosis, malaria and neglected tropical diseases and combating hepatitis, water diseases and other communicable pathologies.” There are great differences in the transmission and in the main groups affected by viral hepatitis, thus, reducing infections and their morbidity and mortality requires a multidisciplinary approach, aligned with the universal health system. Between 1999 and 2020, 689,933 cases of viral hepatitis were reported in Brazil. Of these, 254,389 (36.9%) were hepatitis B and 262,815 (38.1%) were hepatitis C [1].

It is estimated that hepatitis C affects more than 50 million individuals, leading to a higher risk of hepatocellular carcinoma and liver cirrhosis. In 2015, hepatitis C was responsible for 1.34 million deaths, a number that has been growing and is already higher than the number of deaths caused by HIV. Despite the current therapy being able to cure more than 95% of individuals with the infection, the World Health Organization (WHO) recognizes that access to diagnosis and adequate treatment is still insufficient [2].

With the identification of hepatitis C virus more than 3 decades ago, the infection was recognized as a frequent complication of hemodialysis (HD). Risk factors for infection in these patients include number of blood transfusions, duration of HD, and type of dialysis, prevalence of HCV infection in the dialysis unit, previous organ transplant and younger age [3]. The prevalence of HCV infection in the world is around 1%, with wide variation between regions [2]. However, its prevalence was 9.9% in the Dialysis Outcomes and Practice Patterns Study (DOPPS) in 2012–2015, while the incidence was 1.2 new cases per 100 patient-years [4]. Such findings translate into a minimum of 20,000 new cases per year among HD patients. Most infections are nosocomial, resulting from poor hygienic precautions, with a minority acquired outside the HD unit, mainly through the use of intravenous drugs. These numbers are similar with the WHO’s goal of eliminating viral hepatitis by 2030 [5].

In Brazil, in patients undergoing HD, the prevalence tends to be much higher than in the general population (0.5%–0.7%). Data from the 2020 Dialysis Census of the Brazilian Society of Nephrology, carried out with 30% of units in the country, estimate that 2.8% of dialysis patients in Brazil have positive serology for HCV, i.e., 4,000 infected people, out of a total of 144,795 enrolled in dialysis units [6]. In 2013, the prevalence of positive serology for HCV in dialysis units was 4.2%, however a greater decline was expected due to the existence and availability of treatment. Logistical difficulties, access to research and medications, cost and little information may be causes of the low percentage of treatment [2]. Regarding hepatitis B, the prevalence of the virus reduced from 1.4% to 0.7% in the same period [6].

Due to the complexity involved in eliminating the hepatitis C virus, the European Association for the Study of the Liver suggested in 2017 to split the WHO global target into smaller goals. This public policy strategy is known as microelimination, and dialysis is one of the target intervention subgroups. Because there is no vaccine, prevention and treatment are the basis of public health policy to eliminate HCV infections [2, 7]. Thus, the incidence of HCV infection in HD units will drop dramatically due to the reduction in the number of source (infectious) patients, thus creating a healthy behavioral cycle [4].

However, despite the availability of effective and well-tolerated direct-acting antiviral (DAA) treatments [8–10], there is little evidence that the elimination of HCV from HD units is happening. Actions against HCV in these settings can be successful with multidisciplinary collaboration and adequate funding [4]. Initiatives need to be contextualized and disseminated to achieve HCV eradication on HD units, as results convincingly demonstrate that the prevalence of infection can be reduced to a level close to zero in these environments [11]. In this context, it is important to discuss the performance of the multidisciplinary team and nurse-led management in the management of practices that eliminate this transmission [4].

Discussing initiatives considering the micro-elimination process becomes necessary, especially for low- and middle-income countries [12], such as Brazil. Thus, the expansion of scientific productions that point out paths for the microelimination of HCV in HD units, in different scenarios, is justified. The present study aims to analyze the measures to contain the transmission of Hepatitis C in a hospital hemodialysis unit (HDU).

## Methods

### Type of Study

It is a case study composed by multiple units of analysis. Case studies are important for explaining causal links in real-life interventions. These are too complex for experimental or surveys strategies. For this type of study, the properly developed theory must consider the level at which the generalization of results will occur. It is a way of doing empirical research, investigating a current phenomenon within its real context. Therefore, it is an analytical generalization [13].

The prior adoption of theoretical propositions is oriented to conduct data collection and analysis. The quality of the case study is presented by the validity of the construct (detailing the multiple sources of evidence, establishing their chain), internal validity (adequacy to the proposed theoretical standard and the analyzes undertaken) and reliability (related to the structured protocol of research and database consistency) [13].

The explanation is based on cross-analysis of how some situations and events influence others, presenting interactive monitoring of factors in face of their relevant characteristics, incorporating the perspectives of the scenario. In these situations, the most used form of presentation is to have most of the main report contain the cross-analysis, with the individual cases being presented as part of a supplement.

For the present study, the management of the critical event incidence of HCV in HDU by multiple units of analysis was considered. The subunits of analysis were related to: a) analysis of the clinical-epidemiological profile of patients, b) active search for hepatitis cases, c) tracking of transmission routes d) preparation of a management protocol and e) description of the results achieved by the intervention. Table 1 presents the initiatives for conducting the case study and synthesis of the research protocol.

**TABLE 1:** Script for a case study on the management of the critical event incidence of HCV in HDU. Uberaba, Minas, Gerais, Brazil, 2017–2022.

Analysis units	Procedures	Sources	Analysis and reporting plan	Cross analyzes
Critical event management	- Documentary analysis and secondary data, organized in timeline and construction of a protocol	- Records -Records of the unit - Health service documents	- Report containing the variables of interest and document summaries	Critical empirical analysis considering the methodological theoretical framework
Analysis of Subunits				
Analysis of the clinical-epidemiological patients’ profile	Descriptive analysis of secondary data	Patient records	Descriptive analysis of demographic, clinical and laboratory aspects (age, gender, HD time, previous transplant, injecting drug use, HIV patient)	**--**
Active search for hepatitis cases	Descriptive analysis of secondary data organized in a timeline and monitoring of the conglomerate of assisted patients	Patient records	Survey of laboratory results according to current evidence (platelets, aminotransferases and viral load survey)	**-**
Tracing transmission routes	Documentary analysis to report on the itinerary of the critical event and follow-up of the conglomerate of assisted patients	Service records	Identification of organized actions and inappropriate practices described in documents, routines, records of active observation of the care provided and information on employee screening	**--**
Preparation of management protocol	Collective construction for the elaboration of a protocol	Analytical reports and situational analysis committee organization	Creation of the Protocol itself, with the dimensions for the management of the critical event	Elaboration of the Management Protocol as an intervention proposal for the service
Description of the results achieved by the intervention	Descriptive analysis of secondary data organized in a timeline and follow-up of the conglomerate of patients assisted for 36 months	Patient and service records	Descriptive analyzes and surveys regarding laboratory results	The description of the results achieved by the intervention considered the subunits of analysis and presents itself as a strategy for transferring evidence and sharing experience

### Study Scenario

The study scenario is the HDU of a large public and teaching general hospital of the SUS (332 beds) – a reference for the Southern Triangle region of the state of Minas Gerais, Brazil. The HDU works in three daily shifts. The team working in the unit is composed of physicians (*n* = 7) and nurses (*n* = 9), both nephrologists, in addition to nursing technicians (*n* = 16). The turnover of professionals is low, with an average time working together is 15.6 years. The professionals are trained and specialized for the exercise of their activities. It has 12 machines, 11 working on each shift and one specific for isolation. It also has machines for acute cases, hospitalized patients, which are not the population of the present study.

It is noteworthy that the research responds to the WHO guidance for the microelimination of HCV in HD services, as a local initiative. The project was conducted by a research group from the university linked to the teaching hospital studied and was part of a scientific initiation project.

### Study Population

The study population consisted of health service records and medical records, containing information about the assisted patients, unit records, complications, records of active observation for nursing supervision, results of exams performed by employees and other related documents. The period for collecting this information comprised the follow-up on the evolution of patients and the measures adopted since the occurrence of the critical event. It was considered as the moment of perception of the increase in the incidence of HCV among the conglomerate of HD patients, the second half of 2019.

Retrospective and prospective data were analyzed to understand the path of the disease. The study evaluated patient data from the start of dialysis treatment to the present day, after the implementation of the interventions, in July 2022. The gathering of information took place in an intentional and purposeful way to understand the event and its repercussions.

Data extraction was carried out by a group of trained researchers, composed of a nephrologist nurse and four medical students, linked to the teaching hospital and the research group. Data collection was conducted by a guiding script for each analytical subunit between March 2020 and July 2022.

### Study Variables and Analytical Procedures

To understand the critical event and the repercussions of its management and thereby generalize the results, a research protocol was organized for the variables and their corresponding analytical procedures.

For the unit of analysis, the clinical-epidemiological profile of the patients was surveyed: age, sex, time on HD, previous transplantation, or other risk factors such as injecting drug use and being HIV positive. Data were presented descriptively, by absolute and relative frequencies.

As for the active search for hepatitis cases, including occult cases, three criteria were used: presence of reactive serology, high levels of alanine aminotransferase (ALT) and a drop in platelets. It is known that from a biochemical point of view, HCV infection results in an increase in serum ALT levels. Unfortunately, the diagnostic value of the enzyme is quite low in patients with chronic renal disease. Lower values in dialysis patients than in healthy controls have been repeatedly reported [14]. In patients with or without viral hepatitis, their levels are higher in those with intact renal function. The causes of this phenomenon remain unclear. Low nutritional values, high levels of uremic toxins, and the presence of ultraviolet-absorbing materials may explain these differences [15]. As for platelets, it is known that in chronic liver disease with clinically significant portal hypertension, their count drops due to splenomegaly and hypersplenism. However, in chronic renal patients on dialysis, thrombocytopenia is observed with continuous use of heparin.

Therefore, all those who showed a two-fold increase in the previous level of ALT, even within the limit of normality, as well as those who showed platelets below 150,000, should undergo quantification of the nucleic acid of the B and C viruses, even if they had negative serology. These data were presented in a descriptive way. The upper limit of normality for ALT was 19 U/l for women and 30 U/l for men.

Regarding the transmission routes, the service documents were analyzed, as well as the serological screening of employees. For the health worker, as he was immunocompetent, anti-HCV and HBsAg serology was performed. These data were presented in a discursive way, detailing the itinerary of the critical event.

As for the preparation of a management protocol, the protocol itself and the report for its elaboration are presented, which considered the study of the records of the situational analysis committee. The description of the results achieved by the intervention started from the understanding of the phenomenon through the interpretation of the subunits of analysis. The surveillance and management propositions in the hospital HDU, the team’s engagement in evidence transfer strategies, and the sharing of experience through the report were considered.

### Ethical Aspects

In accordance with the Declaration of Helsinki and Brazilian ethical standards, the case study was approved by the Research Ethics Committee of the Universidade Federal do Triângulo Mineiro Hospital, in accordance with Resolution no. 466/2012, which deals with human research (approval No. 3.930.299). Despite the use of secondary data and hospital records, written informed consent was obtained from patients and the coordination of the service for publication of the study.

## Results

### Analysis of the Clinical-Epidemiological Patient’s Profile

The HDU, in August 2019, had 45 patients on HD, 57.8% were men, with a mean age of 50.7 years and a mean duration of HD time of 81.3 months, 11.1% had been previously transplanted, 13.3% had a history of injecting drug use and 6.7% had HIV (Table 2).

**TABLE 2:** Clinical and epidemiological aspects of patients on hemodialysis in a public teaching hospital. Uberaba, Minas Gerais, Brazil, 2017–2022.

Aspects	Patients on hemodialysis	Patients with HCV
Age (years)		
Mean ± SD	50.7 ± 16.0	48.6 ± 12.1
Gender n (%)		
Male	26 (57.8)	5 (100)
Female	19 (42.2)	0
HD time* (months)		
Mean ± SD	81.3 ± 56.3	72.0 ± 19.2
Previous transplant		
Yes	5 (11.1)	0
No	40 (88.9)	5 (100)
Injecting drug use		
Yes	6 (13.3)	3 (60)
No	39 (86.7)	2 (40)
HIV positive		
Yes	3 (6.7)	2 (40)
No	42 (93.3)	3 (60)

Source: Prepared by the author, July/2022.

HD*: Hemodialysis.

During the periodic examinations in the second half of 2019, an increase in cases of hepatitis C among patients was noted in the last 2 years. Therefore, an epidemiological investigation was proposed to identify transmission routes, in addition to the search for occult hepatitis C. The search for occult hepatitis B was performed in the same cases, even if the serological markers were negative.

### Active Search for Viral Hepatitis Cases

The service performs serology for hepatitis B and C every 6 months, as recommended by the Ministry of Health [16]. During the examinations in the second half of 2019 (between August and October), in the exercise of the service’s routine, six of the 45 patients (13.4%) showed reactive anti-HCV and one for HBsAg (2.2%). This critical event aroused the need for epidemiological investigation. Therefore, three criteria for investigation were considered: presence of reactive serology, high levels of ALT and drop in platelets.

The first step was to know the timeline of infections. Of the six patients with reactive anti-HCV, one started dialysis treatment in 2016 and became reactive in the first half of 2017. In the same year, two patients were admitted to the service with positive serology. In 2019, in the laboratory evaluation of the second semester, 3 new cases emerged, all of them already undergoing previous treatment at the service. In the same semester, a patient was admitted to the sector, reactive for HBsAg (Table 3).

**TABLE 3:** Timeline of HCV and HBV infections in a hospital renal care unit. Uberaba, Minas Gerais, Brazil, 2017–2022.

Year/semester	2017/1°	2017/2°	2018/1°	2018/2°	2019/1°	2019/2°
Anti-HCV +	2	3	3	3	3	6
HBsAg +	0	0	0	0	0	1

Source: Prepared by the author, July/2022.

HD*: Hemodialysis.

Between 2019 and 2020, patients were monitored through the analysis of ALT levels at three times: before admission to the service, 6 months after the start of HD and at the time of the beginning of this research.

Of the 45 patients, 23 had, in any of the three dosages, ALT levels above the upper limit of normality or twice the previous value. Six of the 23 patients were cases with positive serology for hepatitis C, who underwent quantification and genotyping of the virus. Therefore, the other 17 cases were selected for HCV-RNA and HBV-DNA quantification, only because of elevation in ALT levels. The case with reactive HBsAg did not have ALT elevation.

Of the six patients reactive for anti-HCV, five had HCV-RNA detection confirmed by the real-time RT-PCR method, produced by the company Abbott (Real Time PCR). The Abbott Real Time HCV Genotype II system (Abbott Diagnostics) was used to determine the HCV genotype based on real-time dual-target PCR: the 5′ UTR target region, the most conserved of the genome, was used to discriminate between HCV genotypes and the NS5B gene was the target of subtyping 1a and 1b. The five patients were diagnosed with genotype 1a of the virus. One case had undetectable HCV-RNA, which has been repeated in subsequent exams, considered a spontaneous cure. Although genotyping was performed at that time to define the therapeutic approach, recently, Glecaprevir/Pibrentasvir, a combination of pangenotypic drugs, proved to be safe and effective in patients with renal dysfunction, being implemented by the Brazilian Ministry of Health. The case with reagent HBsAg had undetectable HBV-DNA in numerous exams. Subsequently, HBsAg became non-reactive, and the initial test was considered to have been a false positive.

Of the five patients who had detectable HCV-RNA, all were men, mean age 44.2 years and mean time from HD 16.4 months to seroconversion. None had been previously transplanted, three had a history of injecting drug use, two of them being treated for HIV. All received treatment for Hepatitis C.

Of the 17 cases with elevation of ALT, ten were submitted to HCV-RNA quantification and eight to HBV-DNA quantification. None of the viruses were detected.

As for platelets, the count was performed in the routine exams immediately prior to the beginning of the research. Of the 45 patients, 15 had a result below the lower limit of normality (<150,000). Only four were not included in the ALT elevation or anti-HCV reagent group. Two performed the quantification of viruses B and C, which were undetectable.

### Tracing Transmission Routes

The alert conditions for a transmission route were identified in the scenario, described below. The need to intensify adequate disinfection of surfaces of beds, rooms and HD machines and organize the medication preparation space was observed. Another routine practice among the team was the reuse of medication for multiple patients, such as heparin. Also, the reprocessing of the capillaries in a collective bench and the inadequate stock of materials were noted.

Also noteworthy is the inadequate adoption of standard precautions by the nursing team, such as failure to change gloves between patients and inadequate hand washing. There was a need for the team to engage in the consistent adoption of precautions, considering that patients may be asymptomatic or have a nonspecific clinical situation. There was a lack of qualification of the information both in the records and during the shift change. Risk behaviors outside the hospital environment could also be relevant, such as injecting drug use. However, adequate screening of new cases that initiate dialysis treatment and treatment of positive ones reduce the spread of the disease.

Another condition involved absenteeism among the nursing team, which resulted in assistance days with a reduced number of employees. Furthermore, adherence to the periodic examination by professionals was an aspect to be reviewed with the team. Considering the critical event, a collective screening was promoted with the unit’s workers, who performed serology for hepatitis B and C, all of which were non-reactive.

A Situational Analysis Committee was created to guide the management of the critical event. Initially, based on determinations by the Ministry of Health and current recommendations on hepatitis C and HD, it was established at the institution that the screening should be conducted by physicians of the institution. Partnerships were made for the clinical management and monitoring of cases, such as consultations between the clinical staff of the HDU and the team of the Gastroenterology service, responsible for the Hepatitis Outpatient Clinic. The other initiatives guided by the committee will be discussed below.

### Preparation of Management Protocol

In 2019, a situational analysis committee was established by internal decree. The committee was composed of a multidisciplinary team of physicians from the HDU and from the gastroenterology discipline, an infectious disease specialist, specialist nurses from epidemiological surveillance and representatives of the patient safety nucleus.

**FIGURE 1:**
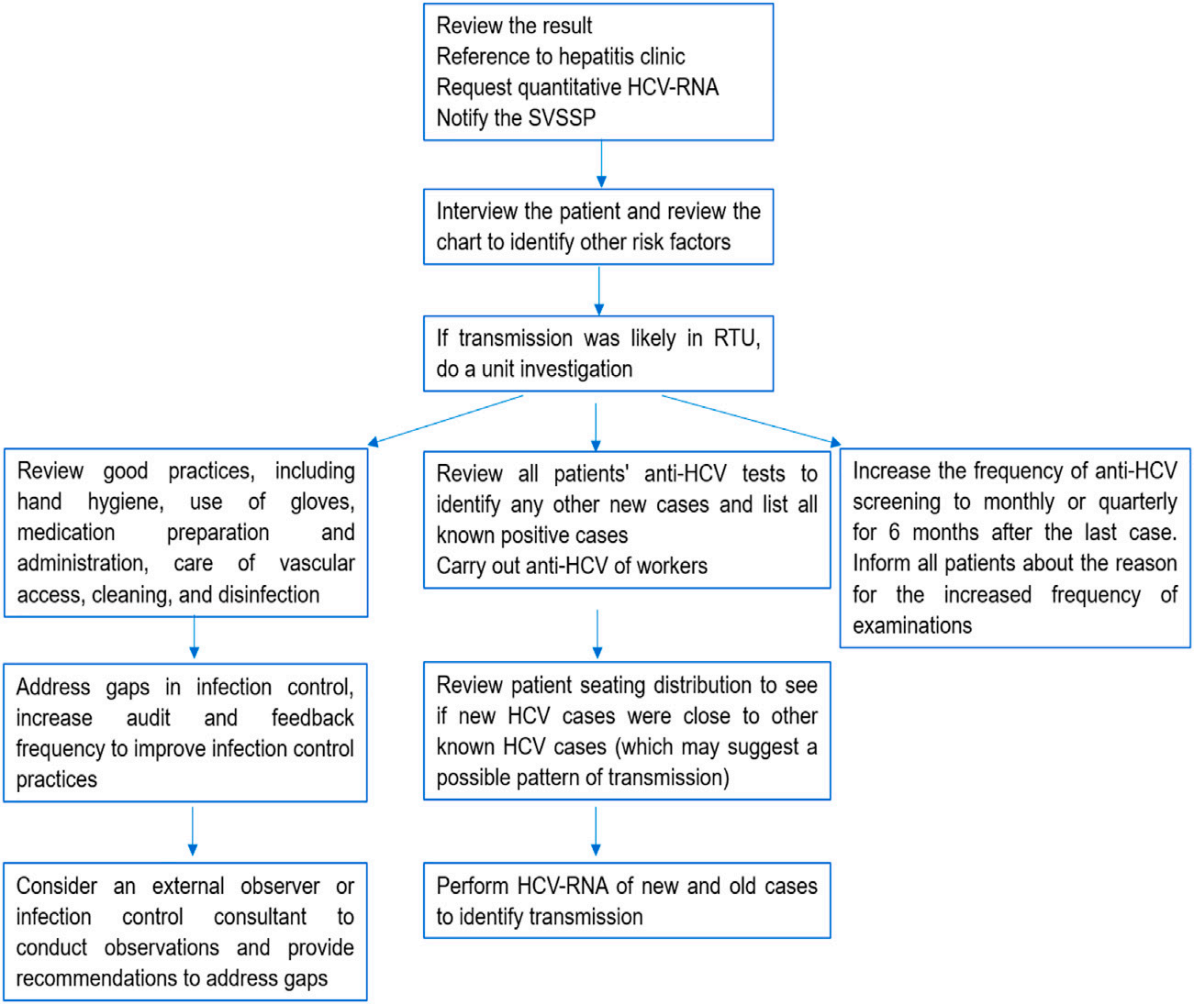
Flowchart for monitoring cases with seroconversion to hepatitis C in HD. Uberaba, Minas Gerais, Brazil, 2017–2022 [17].

The work routine of this committee was based on fortnightly meetings to discuss and monitor the critical event. As a main result, the care protocol for “Control and epidemiological management of seroconversion to hepatitis C in hemodialysis” was created and implemented [17]. The protocol points to purpose, scope of application of routines, target population, legal basis, risk factors for the transmission of hepatitis C in HDU, precautions and preventive measures, screening for hepatitis C in HDU, notification system and monitoring flowchart of cases with seroconversion to hepatitis C on HD, in addition to monitoring by the care team (Figure 1). Its dissemination took place through publication on the hospital’s website, with open access, where other care protocols are available. Awareness actions were also carried out with the HDU professionals through meetings and an individual approach with each worker.

### Description of the Results Achieved by the Intervention

The hospital HDU of this study was monitored during the 3 years following the start of the project. The five patients with HCV were treated and the disease was eradicated, as demonstrated by the absence of HCV-RNA at weeks zero, 12, and 24 after the end of treatment, and every 6 months thereafter (Table 4).

**TABLE 4:** List of patients with HCV, RNA-HCV values, Genotyping, Treatment type and HCV-RNA at follow-up. Uberaba, Minas Gerais, Brazil, 2017–2022.

Patients	HCV RNA (UI/mL)	Genotype	Treatment	RNA in follow-up
A	156,546	1a	Glecaprevir/Pibrentasvir 300/120 mg	Undetectable
B	452,722	1a	Sofosbuvir/Velpatasvir 400/100 mg	Undetectable
C	444,041	1a	3D/Ribavirina (12.5 + 75+50)/250 mg	Undetectable
D	301,693	1a	3D/Ribavirina (12.5 + 75+50)/250 mg	Undetectable
E	1,897,461	1a	3D/Ribavirina (12.5 + 75+50)/250 mg	Undetectable

Source: Prepared by the author, 2022.

HD*: Hemodialysis.

A nephrologist nurse started to manage the cases, with screening for hepatitis C in all patients who start the dialysis program or are transferred from other centers. Monthly monitoring of ALT levels and semiannual serology for hepatitis B and C were strengthened. In cases of increased ALT levels and/or positivity for anti-HCV, a molecular biology test is performed for the detection of HCV-RNA. If there is seroconversion, the screening of susceptible individuals is immediately carried out in the unit, including of the care team. Then, the anti-HCV is performed monthly for 3 months in all vulnerable to the disease, then quarterly and after that, it returns to the biannual routine. Once the patient is reactive for anti-HCV, even after the virus has been eradicated, he is submitted to a molecular biology exam every 6 months, given the risk of reinfection.

The engagement of nursing supervision in the adoption of precautions and preventive measures in the work routine must be reported, which were reinforced with the team. There was active observation of practices, close monitoring of workers, and immediate correction of the techniques performed.

The correct form of preparation and administration of medications during HD was reviewed, as well as the procedures for adequate disinfection of beds, rooms, and machines. The proper way of using disinfectants was reinforced, as well as time required for decontamination.

Aiming to reach all the people who circulate in the unit (workers, teachers, and students), the crucial moments related to hand hygiene were emphasized. They are before and after contact with the hemodialysis catheter, and with the needles for puncture of the arteriovenous fistula; in the measurement of vital signs and capillary blood glucose; after removing gloves; before and after handling medications; immediately after exposure to bodily fluids; after touching patient contact surfaces.

There was institutional engagement, expressed by the organization of the Situational Analysis Committee and the preparation of the care protocol. Since then, no new cases have been detected, which denotes the adoption of the protocol for “Epidemiological control and management of hepatitis C seroconversion on hemodialysis,” as well as reflecting the multidisciplinary efforts in the conduction and management of the critical event.

## Discussion

The present study is a report of an innovative initiative for the management of the critical event regarding the increase in the incidence of HCV infection in hospital HDU. The incidence of hepatitis C in dialysis units has been considered a challenge for the elimination of the virus, requiring innovative ways to organize health services. Although several micro-elimination programs have been developed around the world, few initiatives target high-risk populations in health services. On the other hand, healthcare-associated HCV infections are known to be prevalent, particularly in low- and middle-income countries [18].

Regarding the clinical-epidemiological profile, a systematic review with meta-analysis that aimed to analyze the risk factors for HCV infection in dialysis patients, showed a positive correlation between HD time and the rate of HCV infection (*p* < 0.01). HD patients, especially from Asia, using shared machines, undergoing dialysis sessions more than twice a week, receiving blood transfusions, previously undergoing kidney transplantation and drug-dependent were at risk of HCV infection. The rate of HCV infection increased with the duration of HD [19]. Although the case study descriptively analyzes the clinical and epidemiological aspects, the results described here correspond to the reality identified about the aspects and risk factors for patients on HD.

The present study found five patients with active HCV infection in the HDU in question. According to the reported results and according to the specialized literature, it is believed that the incidence of HCV infection in HD will drop dramatically after reducing the number of source (infectious) patients. Infection control guidelines point to mandatory initial screening for HCV on admission to the unit, in addition to periodic testing [18]. ALT values should be analyzed carefully, their abnormality being indicative for virus screening [19].

The current challenge is to test all patients at the beginning of dialysis, as the prevalence of HCV at the beginning of HD is around 5% in countries with relevant DOPPS data (United States, United Kingdom, Germany, Italy, Spain and Japan), substantially higher than in the general population [2]. Patients with positive anti-HCV antibodies at the start of dialysis should be treated promptly and this would avoid isolation policies for HCV-positive patients as those used in Taiwan [4] and several other countries [2]. Isolation is costly, did not prove to be effective and is not recommended by the CDC or the KDIGO guideline [10, 12]. It is important to note that the testing policy at the beginning of HD should not prevent the testing of patients who return to the service after periods of treatment in another location, especially in a country with the highest prevalence [11], as well as in those with risk factors for acquiring HCV outside the dialysis context.

A crucial aspect is bridging the gap in awareness and motivation of nephrologists for the care of HCV in dialysis facilities. In this context, the collaboration between gastroenterologists and nephrologists is denoted as an action for the active search of cases and reduction of the incidence of HCV in the HDU [18].

Regarding nosocomial transmission routes, the reality reported in this case study is similar to a lesser extent to that discussed in scenarios of Pakistan [3] and China [18], where patients had more opportunities to be exposed to medical equipment, objects or hands contaminated by HCV of medical personnel.

The application of institutional protocols of good practices in patient care is essential for the microelimination of hepatitis C in HD. According to the results of the present case study, relatively simple measures should be encouraged by health establishments and correctly used by their professionals, thus aiming at the elimination and/or mitigation of risks arising from care practices. Despite this reality being challenging, often due to the lack of adequacy between theory and practice, lack of adequate materials and relaxation of some professionals regarding their self-care, the development and application of the protocol resulted in the elimination of the hepatitis C virus in the unit. Such mobilization demonstrated the success of the measures adopted [20].

No experience reports or case studies were identified in the Brazilian literature that address the management of the incidence of hepatitis C in hospital HDUs through institutional actions that involve the organization of a situational analysis committee and the development and application of a protocol, considered here as essential strategies to achieve the elimination of the virus from the study scenario.

### Study Limitations

As for the limitations, the case studies are not subject to a generalization other than their analytical understanding of theories and scenarios. However, it is a method considered very useful when the phenomenon cannot be analyzed outside the context in which it naturally manifests itself [13].

The present study demonstrates strategies for the microelimination of the C virus in a dialysis environment, corroborating the WHO guidelines for the eradication of hepatitis C by 2030. It is still relevant for contributing to future research that expands methodologies for service evaluation regarding the implementation of critical event management strategies. And it presents itself as a resource for professional training and organization of services by detailing the measures to contain the transmission of Hepatitis C in a hospital HDU.

### Conclusion

The report described exemplifies a path towards the eradication of HCV from HD units, which is considered urgent worldwide. Even considering the limitations of a case study, the present research becomes relevant for pointing out the measures in the management of this critical event. With the analysis of the clinical-epidemiological profile of the patients, the active search for hepatitis cases, the tracking of transmission routes, the preparation of a management protocol and it is possible to transfer the evidence obtained, sharing the entire experience of the project. Also, the study, due to the success achieved in eradicating the virus in the unit, constitutes as a reference for the orientation of services and for the multidisciplinary training, especially on how to guide the integrated action between nephrologists and gastroenterologists.
